# TAp73 is one of the genes responsible for the lack of response to chemotherapy depending on B-Raf mutational status

**DOI:** 10.1186/1479-5876-8-15

**Published:** 2010-02-10

**Authors:** Marta Herreros-Villanueva, Pilar Muñiz, Carlos García-Girón, Mónica Cavia-Saiz, María J Coma del Corral

**Affiliations:** 1 Unidad de Investigación, Hospital General Yagüe, Burgos, Spain; 2 Departamento de Bioquímica, Universidad de Burgos, Burgos, Spain; 3 Servicio de Oncología, Hospital General Yagüe, Burgos, Spain

## Abstract

**Background:**

Although there have been many studies on the p73 gene, some of its functions still remain unclear. There is little research on the relationship between p73 gene transcription and its protein expression and the response to certain drugs such as oxaliplatin and cetuximab, which are drugs currently used in colorectal cancer.

The purpose of this study was to evaluate the impact of TAp73 expression on oxaliplatin and cetuximab-based chemotherapy in colorectal cancer cell lines with different K-Ras and B-Raf mutational status.

**Methods:**

TAp73 was analyzed in three colorectal tumor cell lines HT-29, SW-480 and Caco-2. mRNA TAp73 was determined using Real time PCR; TAp73 protein by immunoblotting and cell viability was analyzed by the MTT method.

**Results:**

We found that mRNA and TAp73 protein were decreased in cells treated with oxaliplatin (in monotherapy or combined with cetuximab) when B-Raf is mutated. This was statistically significant and was also associated with higher cell viability after the treatment.

**Conclusions:**

Here, for the first time we report, that there is a signaling loop between B-Raf activation and p73 function.

Low expression of TAp73 in colorectal cancer cell lines with mutated B-Raf may be involved in the lack of response to oxaliplatin in monotherapy or combined with cetuximab.

## Background

The incidence of colorectal cancer has been increasing in the last few years, while the age of diagnosis is decreasing, and today it is the third or fourth cause of death in the world. The treatment of metastatic colorectal cancer (mCRC) has changed drastically since the 1980s, when only fluorouracil (5-FU) was available for treatment and the median survival was at the most 12 months, to a time when mCRC is considered more of a chronic disease in which the median survival is now reported to be in excess of 2 years, although the 5-year survival rate is still less than 10% [1]. The advances in the treatment of this disease include studies of single-agents vs. combination treatment with 5-FU/leucovorin, irinotecan, oxaliplatin, and capecitabine, and the role of targeted agents such as cetuximab and bevacizumab.

The platinum-based chemotherapy drugs cisplatin, carboplatin, and oxaliplatin are among the most active and widely used agents for the treatment of colorectal cancer today [2]. Cisplatin is a third-generation platinum compound and like the rest of these agents, (oxaliplatin) kills tumor cells primarily by causing DNA damage [3].

Over the last few years, it has been reported that colorectal cancer is a polygenic disease in which oncogene mutation activation and tumor suppressor gene inactivation play important roles in the development of the disease and in the response to the chemotherapy.

### P73

*TP73 *is a gene that was described by Kaghad et. al. in 1997 [4] and is a family member of the tumor suppressor gene *TP53*. *TP53 *and *TP73 *share significant structural and functional homology. Both genes contain an NH_2 _terminal transactivation domain, and a COOH-terminal oligomerization domain, and are capable of inducing cell cycle arrests and cell death in response to DNA damage. However, there is some evidence that shows that the roles of *p53 *and *p73 *in human tumor genesis are different.

*P73 *contains carboxy-terminal spliced variants known as the TA isoforms. The So-called ΔN variants also exist, which lack the transactivation domain and are transcribed from an internal promoter within exon 3 of the full-length genes [5]. These different isoforms have been shown to have vastly different activities. The TA isoforms act similarly to p53, inducing apoptosis. In comparison, ΔN isoforms have little transactivation activity and play a role blocking target genes of *p53 *and their respective *TAp73 *isoforms [6]. Therefore, the TA isoforms may be expected to have functions in tumor suppression while ΔN isoforms might be oncogenic.

For the first time in 2006, Dominguez et al. demonstrated an association between upregulation of Δ*TAp73 *isoforms and poor prognosis in colorectal cancer, specifically advanced tumor stage, suggesting that they may be of practical clinical prognostic value [7]. Last year, some authors also demonstrated that high expression of *TAp73 *in colorectal cancer may be involved in the progression of colorectal cancer and may serve as a potential index to predict differentiation level and prognosis of colorectal cancer [8].

Although there are many reports concerning the *p73 *gene, some of its functions remain unclear. Little research has been reported on the relationship between p73 gene transcription and its protein expression with the response to certain drugs such as oxaliplatin and cetuximab which are drugs currently used in colorectal cancer.

Epidermal Grown Factor Receptor (EGFR) is one of the most important cell membrane receptors expressed in normal cells [9]. The EGFR molecular structure includes an extra-cellular region, a transmembrane domain and a protein tyrosine kinase region [10]. Epidermal Grown Factor (EGF) is a natural ligand of EGFR.

EGFR is abnormally activated in many epithelial tumors and it is frequently over expressed in colon cancer, correlating with a poor response to treatment, disease progression and poor survival [11].

In the early 80s the EGFR was pointed out as a potential target for cancer therapy [12] and two anti-EGFR strategies were adopted: monoclonal antibodies (Mabs), which bind the extracellular domain, interfering with the natural ligand, and low-molecular-weight tyrosine kinase inhibitors, which interfere with the tyrosine kinase domain [13]. Cetuximab is a chimeric monoclonal antibody antagonist for EGFR that binds to EGFR with high affinity and prevents the ligand from adopting the conformation for dimerization and activation [14-17].

The most important mediators in EGFR signaling are K-RAS and B-RAF kinase proteins. Mutations in these effectors have been found in various cancers [18,19].

*K-Ras *and *B-Raf *mutations are found in up to 50% and 10%, respectively of colon cancers and appear relatively early in the carcinogenesis pathway leading to constitutive activation of its proteins [20,21]. Upon activation, RAS recruits RAF protein to the cell membrane and binds it directly, activating RAF kinase. B-RAF is considered to be the principal RAF isoform linking *Ras *to MEK signaling.

Several studies have indicated that the presence of mutant K-Ras in colorectal cancer correlates with a poor prognosis [21-23] and is associated with lack of response to EGFR inhibitors such as cetuximab [24,25]. Wild type *K-Ras *status is currently required to administer cetuximab in monotherapy, or combined with other agents, as it has been demonstrated that this is necessary but not sufficient to confer sensitivity to Cetuximab [26]. Some authors have recently concluded that B-Raf wild-type is also required for response to cetuximab and could be used to select patients who are eligible for the treatment [27]. However, not all of the wild type *K-Ras *and *B-Raf *patients are responding to cetuximab.

Therefore, the identification of additional genetic determining factors of the action mechanism of EGFR-targeted therapies in colorectal cancers (CRCs) is important at least for two reasons. First, the understanding of the molecular basis of therapies could allow the rational design of alternative treatment strategies. Second, to prospectively identify patients who should not receive either treatment, this way avoiding their exposure to ineffective and expensive therapy.

As it is well known P73 cooperates with Ras in the activation of MAPK kinase signaling cascade [28], we investigated the relationships between TAp73 expression and *K-Ras/B-Raf *status as regards of the chemosensitivity. Currently there are no data published on the correlation between TAp73 and cetuximab. In an attempt to further characterize this complex pattern of expression in human colorectal cancer cell lines and to assess its role in response to chemotherapy, the purpose of this paper was to analyze TAp73 mRNA and TAp73 protein expression in colorectal cancer cell lines treated with cetuximab and oxaliplatin, using Real Time PCR and Western Blot to explore associations between *p73 *expression and *K-Ras/B-Raf *status.

For the experimental model of our study, we chose three human colon cancer cell lines: HT-29, SW-480 and Caco-2. These enterocyte cell lines were derived from human primary colon adenocarcinomas and are established cell models for the study of the biology and drug treatment of cancer. These cells lines are different in K-RAS and B-RAF pathways, as HT-29 harbors the V600E *B-Raf *heterozygotic mutation [29], SW-480 which harbors *K-Ras *mutation and Caco-2 is *K-Ras *and *B-Raf *wild type.

The association between the expression of *TAp73 *and the presence/absence of *K-Ras *and *B-Raf *mutations in response to cetuximab supports their possible apoptotic function and helps to understand the action mechanism of this drug.

## Methods

### Tumor cell lines and culture conditions

HT-29, SW-480 and Caco-2 human colorectal carcinoma cell lines were obtained from American Tissue Culture Collection (ATCC). All tumor cell lines were maintained in Dulbecco's minimal essential medium (DMEM) supplemented with 5% fetal bovine serum, 2 mM L-Glutamine, 100 U/mL penicillin and 100 mg/ml streptomycin. Cells were maintained at 37°C in a 5% CO_2 _incubator in monolayer culture to 75% to 90% confluence and detached using 0.05% trypsin-EDTA.

Cells were counted using trypan blue and were adjusted to the desired concentration for plating.

### Reagents and drugs

Cetuximab (C225, Erbitux^®^) was purchased from Merck Serono and Oxaliplatin from Ratiopharm. DMSO vehicle control was included in all the experiments.

Cells were plated in 25 cm^2 ^culture flasks (Becton Dickinson) at 7.5 × 10^5 ^cells per flask and incubated for 24 hours. After the cells were attached, Oxaliplatin, Cetuximab, both of them, or drug control were added at the concentrations indicated and incubated for 48 hours at 37°C. The concentrations were 10 nM Cetuximab (recommended concentration by Merck and the most used concentration used in the literature) and 5 μM Oxaliplatin (also the most frequent concentration used in the literature).

### Cell-viability assay

Cell growth was determined using a MTT assay as previously described [30]. Human colon cancer cells were cultured in a 96-well plate (Becton Dickinson) at density of 5 × 10^4 ^cells per well. The cells were then treated with fixed concentrations of oxaliplatin, cetuximab or both drugs. After 24, 48 and 72 h, the cells were treated with MTT (Sigma-Aldrich). Plates were incubated in the dark for 4 h, and the absorbances were measured at 570 nm using a microtiter plate reader (Bio-Tek). To determine cell viability, percent viability was calculated as [(absorbance of drug-treated) sample/(control absorbance)] × 100.

### RNA isolation and Real Time PCR analysis

Total RNA was extracted with TRI reagent (Ambion) following the manufacturer's protocol. cDNA was prepared using SuperScript™ II First-Strand Synthesis System for RT-PCR (Invitrogen) according to the manufacturer's protocol. The sequences of the primers used for PCR were as follows: TAp73-Forward: 5'-GCACCACGTTTGAGCACCTCT-3'; TAp73-Reverse: 5'-GCAGATTGAACTGGGCCATGA-3'. The reference gene used to standardize expression results was Ubiquitin C (UBC): UBC-Forward: 5'-ATTTGGGTCGCGGTTCTTG-3' and UBC-Reverse: 5'-TGCCTTGACATTCTCGATGGT-3'. Set primers were all as described previously [31].

Real-time PCR was performed in a final reaction volume of 50 μl containing 25 μl of 2× SYBR Universal PCR Master Mix (Applied Biosystems), 0.5 μM/L of each primer and 4 μl of cDNA. PCR was performed in MicroAmp optical 96-well plates with optical adhesive covers (Applied Biosystems). Amplification and detection were performed with an ABI prism 7500 sequence detection system (Applied Biosystems). The amplification conditions were 2 minutes at 50°C and 10 minutes at 95°C for AmpliTaq Gold activation, followed by 40 cycles of 15 seconds at 95°C for denaturation and 1 minute at 60°C for annealing and extension. The specificity of each primer set was confirmed by melting curve analysis.

### Western Blot Analysis

For protein analysis, 7.5 × 10^5 ^cells were seeded, and after treatment, harvested, washed in 1 ml of cold PBS and lysed in EBC lysis buffer (50 mM Tris pH8, 120 mM NaCl, 0.5% NP-40) supplemented with a cocktail of protease inhibitors (Roche). Immunoblots were performed as described previously [32] and incubated overnight at 4°C in the following primary antibodies: mouse anti-p73 Ab-2 and Ab-4 1:500 (Oncogene) and rabbit anti-actin AA20-33 1:5000 (Sigma-Aldrich). Membranes were incubated with the appropriate HRP-coupled secondary antibodies (Pierce) and the enhanced chemiluminescence was detected with Super Signal West-Pico Chemiluminescent Substrate from Pierce. The protein expression levels were measured in a GS800 densitometer and using Quantity-One 4.6.8 Analysis Software (Bio-Rad).

### Data analysis

The mRNA levels expression was determined by relative quantification using the comparative threshold cycle method (2^-ΔΔCT ^Method), described and validated previously [33-35] Each sample is run in quadruplicate and the cell assays were made in triplicate. We validated this assay analyzing several controls (Untreated cells and genomic DNA from Applied Biosystems). In addition a melting curve analysis was performed which resulted in single product specific melting temperatures as follows: UBC, 81.8°C and TAp73, 84.5°C. No primers-dimers were generated during the applied 40 real-time PCR amplification cycles.

### Statistical Analysis

Results are presented as means and standard deviation (SD), and P < 0.05 was considered statistically significant. Statistical analysis was performed with SPSS 11.0 (SPSS, Chicago, IL) for Microsoft Windows XP (Redmond, WA). The paired Student t test (2-tailed) was used to compare the values between treated and untreated cells and Anova test to compare the values among the three lines of cells.

## Results

We characterized HT-29, SW-480 and Caco-2 cell lines according to their viability, mRNA and protein TAp73 expression. We evaluated the role of TAp73 in untreated and treated conditions in order to compare their behavior and correlate their gene expression profile changes with K-Ras and B-Raf status.

### Cell viability assay

HT-29 was compared to SW-480 and Caco-2 regarding cell growth under normal conditions (only treated with vehicle drug) at 24, 48 and 72 hours and after treatment with oxaliplatin, cetuximab and both.

**Figure 1:**
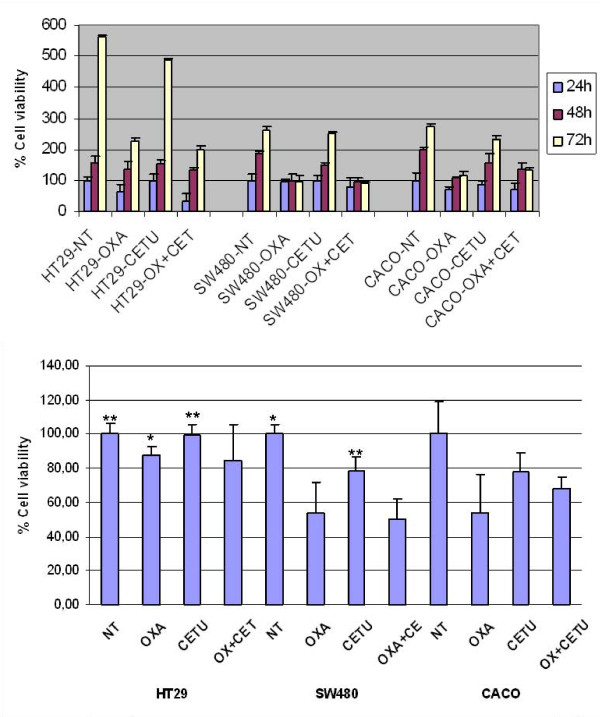
**HT-29, SW-480 and Caco-2 viability assay**. (A) Viability assay at 24, 48 and 72 hours. Untreated (NT), 5 μM Oxaliplatin (Oxa), 10 nM Cetuximab (Cetu) and 5 μM Oxaliplatin plus 10 nM Cetuximab (Oxa+Cetu). Cell grown was determined using a MTT assay. (B) Viability assay after 48 hours of treatment. T-Student analysis. *P < 0.05 **P < 0.01. Each point represents a mean of triplicate values for each sample ± SD.

The viability percentage of the untreated cell lines at the time of 24, 48 and 72 hours are showed in Figure 1a and p-values in Additional File 1. In absence of the treatment, the percentage of viability at 72 hours of the cells HT-29 was higher than in SW-480 and Caco2. This result is correlated with B-Raf mutational status as HT-29 harbors V600E mutation while SW-480 (which harbours K-Ras mutation) and Caco-2 (K-Ras wild type) are B-Raf wild type. This data confirm that B-Raf could confer greater viability than a wild genotype in colorectal cancer cell lines.

The treatment at 24 hours only affects to the viability of Caco-2 cells treated with oxaliplatin alone or plus cetuximab where we observed a significant decreased compared with the control group. In contrast, the treatment for 48 hours decreases the cell viability in all cell lines, being this decrease significative for the treatment with oxaliplatin alone or combined with cetuximab in the SW-480 and Caco-2 cells, and with cetuximab in monotherapy in the SW-480 (Figure 1b). After 72 hours, a decrease in the viability percentage was observed only when the cells were treated with oxaliplatin in monotherapy. No changes were observed in presence of cetuximab in monotherapy and the combination oxaliplatin only affect to the HT-29 and Caco-2 cells.

The treatment effect on viability percentage when comparing the different cell lines, is shown in Table 1. The result shows that there are significant changes among the three cell lines at 24 and 48 hours of treatment. However, at 72 hours we only observed significant differences in the untreated cells and treated with oxaliplatin plus cetuximab.

**Table 1:** Comparative study of the percentage of viability among Caco-2, SW-480 and HT-29 cell lines at different time of treatments.

Time	Treatment	Caco-2	SW-480	HT-29	P value
**24 H**	NT	0.72 ± 0.07	1.30 ± 0.23	0.80 ± 0.17	0.012
	
	OXA	0.51 0.09	1.22 ± 0.11	0.58 ± 0.05	< 0.001
	
	CETU	0.67 ± 0.12	1.27 ± 0.20	0.59 ± 0.16	0.004
	
	OXA+ CETU	0.29 ± 0.05	1.03 ± 0.28	0.57 ± 0.10	0.006

**48 H**	NT	1.29 ± 0.24	2.36 ± 0.13	1.22 ± 0.07	<0.001
	
	OXA	0.73 ± 0.15	1.31 ± 0.22	1.08 ± 0.05	0.012
	
	CETU	1.03 ± 0.11	1.88 ± 0.15	1.28 ± 0.41	0.017
	
	OXA+ CETU	0.91 ± 0.06	1.32 ± 0.13	1.05 ± 0.20	0.032

**72 H**	NT	3.48 ± 0.02	3.23 ± 0.40	2.02 ± 0.11	0.017
	
	OXA	1.44 ± 0.13	1.19 ± 0.25	0.89 ± 0.07	0.100
	
	CETU	3.03 ± 0.15	3.13 ± 0.11	2.43 ± 0.31	0.079
	
	OXA+ CETU	1.55 ± 0.15	1.26 ± 0.03	1.00 ± 0.08	0.025

### mRNA *TAp73 *expression

In order to investigate if the increase in cell viability associated to *K-Ras *and *B-Raf *mutation after the treatment was mediated by *p73*, we analyzed the apoptotic *TAp73 *isoforms.

**Figure 2:**
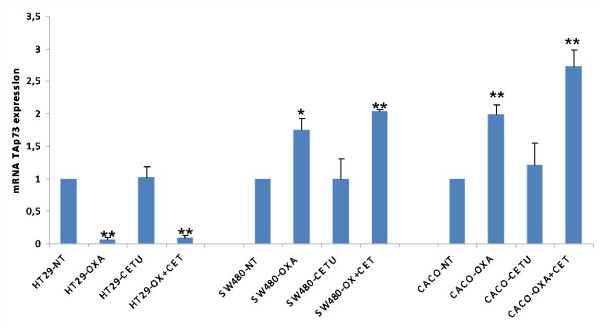
**mRNA TAp73 expression after 48 hours of treatment**. Untreated (NT), 5 μM Oxaliplatin (Oxa), 10 nM Cetuximab (Cetu) and 5 μM Oxaliplatin plus 10 nM Cetuximab (Oxa+Cetu). T-Student analysis. *P < 0.05 **P < 0.01. Each point represents a mean of triplicate values for each sample ± SD.

Relative quantification using Real Time PCR was performed to determine the influence of chemotherapy in mRNA *TAp73 *expression depending on the *K-Ras *and *B-Raf *status after 48 hours of treatment (Figure 2). p-values are showed in Additional File 2.

This analysis showed us that in HT-29 cells, the treatment with oxaliplatin and oxaliplatin plus cetuximab dramatically decreased mRNA *TAp73 *levels. There were statistically significant differences between untreated cells and those treated with oxaliplatin in monotherapy or oxaliplatin plus cetuximab.

In comparison, in SW-480 and Caco-2 cells treated with oxaliplatin in monotherapy or in combination with cetuximab, increasing mRNA *TAp73 *levels were observed. In these cells there were statistically significant differences between untreated cells and those treated with oxaliplatin and oxaliplatin plus cetuximab.

While, regardless of the *K-Ras *and *B-Raf *mutational status, cetuximab in monotherapy has no impact on mRNA TAp73 expression, oxaliplatin alone or in combination with cetuximab induces significant changes in *TAp73*. With these data, we believe that B-Raf mutational status may be one of the genes responsible for the changes in mRNA *TAp73 *expression levels. After treatment with oxaliplatin in monotherapy, or in combination with cetuximab, *B-Raf *mutation induces repression of mRNA *TAp73*.

### Protein TAp73 expression

Immunoblot assays were performed to determine whether mRNA *TAp73 *levels were directly responsible for reduced or increased levels of TAp73 protein.

**Figure 3:**
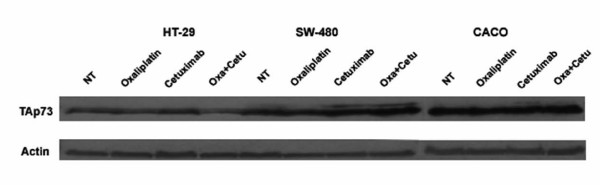
**Protein TAp73 expression after 48 hours of treatment**. Untreated (NT), 5 μM Oxaliplatin (Oxa), 10 nM Cetuximab (Cetu) and 5 μM Oxaliplatin plus 10 nM Cetuximab (Oxa+Cetu). Immunoblot analysis of TAp73 isoforms was performed 48 hours after treatment. Actin expression was used as loading control.

When measuring TAp73 by western blot and protein expression levels in a densitometer (Quantification values are showed in Additional File 3), it was observed that in untreated cells, Caco-2 expressed significantly higher (p < 0.005) levels of TAp73 protein than SW-480 and HT-29 cells (Figure 3). These data suggest that TAp73 could be one of the many downstream RAS/RAF/ERK proteins that could be modulating the apoptosis induced by chemotherapeutic agents, as when *K-Ras *and *B-Raf *are wild type, cells are more sensitive to apoptosis induced by these drugs.

These findings could corroborate the data published by other authors showing that p73 is a determinant of chemotherapeutic efficacy in humans [36].

In HT-29 cells, it was found that after 48 hours, the treatment with oxaliplatin and oxaliplatin plus Cetuximab came out in a decreased TAp73 protein, reaching minimal levels (Figure 3). In this case, a direct correlation between mRNA and protein levels was obtained.

TAp73 protein levels were increased in SW-480 and Caco-2, when these cells were treated with cetuximab in monotherapy, and with oxaliplatin plus cetuximab. As the RT-PCR primers and antibody used were specific to TAp73, it is believed that cetuximab could induce a post-transcriptional regulation process in TAp73 expression.

The results of TAp73 protein expression after 72 hours of treatment were similar to those at 48 hours (data not shown).

When looking at oxaliplatin, it can be concluded that when B-Raf is wild type (regardless of K-Ras mutation), increased levels of p73 protein correlate enhanced TAp73 transcription, in the presence of cetuximab (cetuximab or cetuximab plus oxaliplatin).

When B-Raf is mutated, TAp73 mRNA levels correlate with reduced protein levels.

## Discussion

P73 were cloned due to their structural similarity to *p53 *and have been shown to share functions with the tumor suppressor gene *p53*, but their contributions to the inhibition of tumor formation or to the response to chemotherapy has been uncertain. Many studies have revealed p53-like functions of TAp73, such as their ability to induce apoptosis, yet initial studies indicated that *p73 *were not often mutated in human cancer [5].

It is known that abnormal expression of *p73 *gene plays an important role in the progression of colorectal cancer and its detection may be used to predict the prognosis of colorectal cancer and to guide treatment [8].

P73 has long been recognized as central to the induction of apoptosis in response to DNA damage, a function thought to be critical for tumor suppression and the response of tumor cells to chemotherapy agents [37].

Previous results suggest that p73 contributes to chemotherapy-induced apoptosis and support a model where p53 mutations induce chemoresistance, at least partly, through neutralization of p73 [36]. In this paper, we report for the first time that B-Raf mutations could also be increasing resistance to chemotherapy.

We explored the association of p73 expression levels as regards *K-Ras *and *B-Raf *status with the response to chemotherapy treatments in colorectal cancer cell lines. Our results indicate that, regardless of K-Ras mutational status, TAp73 is induced by oxaliplatin (in monotherapy or in combination with cetuximab) when B-Raf is wild type. On the contrary, *B-Raf *mutations inhibit the transcriptional activation of TAp73 induced after oxaliplatin treatment.

We came to the conclusion that if TAp73 is regulated differently depending on the *B-Raf *status, this could be a good reason for the lack of response to chemotherapy when *B-Raf *is mutated. When *B-Raf *is mutated, the cells showed higher viability than *B-Raf *wild type cells. These data confirm that *B-Raf *mutations could confer a more aggressive tumorigenic phenotype than *K-Ras *while it could be inducing chemoresistance. We also observed that *K-Ras *mutation confers greater viability than a wild genotype in colorectal cell lines.

In our model it was difficult to correlate the TAp73 gene expression profile and protein expression after cetuximab treatment. We speculate that some p73 isoforms (TA or DN) could exert negative post-transcriptional effects leading to different mRNA stability in other p73 isoforms. Similar mechanism was described studing Myc regulation in neuroblastoma cells [38].

It is possible that the interaction between the family members and their isoforms may prove to be an extremely important aspect of chemotherapy response. In this sense, there is evidence that the interaction between p53, p73 and p63 may be involved in the response to this drug. Further experiments will be necessary to clarify this point.

In this case, we found a close correlation and specificity of mRNA *TAp73 *expression with the oxaliplatin and cetuximab response, suggesting that this method is useful to analyze the TAp73 profile dynamics.

## Conclusion

Oxaliplatin in monotherapy or in combination with cetuximab produces an mRNA and protein TAp73 regulation effect. This effect is different depending on *K-Ras *and *B-Raf *mutational status, as we observed in HT-29, SW-480 and Caco-2 models.

When *B-Raf *is mutated, oxaliplatin induces TAp73 downregulation, while when *B*-*Raf *is wild type, the treatment induces TAp73 upregulation. This induction is maintained when the treatment is combined with cetuximab.

We report, for the first time, that *B-Raf *mutations could confer a more aggressive tumorigenic phenotype than *K-Ras*, and could be inducing chemoresistance.

## List of Abbreviations

B-Raf: V-raf murine sarcoma viral oncogene homolog B1; DMSO: Dimethyl sulphoxide; K-Ras: human homolog of the Kirsten rat sarcoma-2 virus oncogene; EGFR: Epidermal Grown Factor; EGFR: Epidermal Grown Factor Receptor; 5-FU: Fluorouracil; MTT: Thiazolyl Blue Tetrazolium Bromide; mCRC: metastatic colorectal cancer; TAp73: transcriptionally active p73.

## Conflicting interests

The authors declare that they have no competing interests.

## Authors' contributions

MH carried out experimental design and molecular genetic study and drafted the manuscript. PM participated in the design of the study and drafted the manuscript. CG carried out experimental design. MC carried out cell culture experiments. MJ participated in the study design and coordination. All the authors read and approved the final manuscript.

## Supplementary Material

Additional file 1 **p values in viability assays**. P values corresponding to HT-29, SW-480 and Caco-2 after 24, 48 and 72 hours after treatment. Related to Figure 1. Click here for file

Additional file 2 **p values in mRNA TAp73 expression**. P values corresponding to mRNA TAp73 expression after 48 hours of treatment. Related to Figure 2. Click here for file

Additional file 3 **Protein expression levels**. Arbitrary Units corresponding to the protein expression levels measured by densitometry. Click here for file
